# Machine learning-based predictive and risk analysis using real-world data with blood biomarkers for hepatitis B patients in the malignant progression of hepatocellular carcinoma

**DOI:** 10.3389/fimmu.2022.1031400

**Published:** 2022-12-12

**Authors:** Yuemin Nan, Suxian Zhao, Xiaoxiao Zhang, Zhifeng Xiao, Ruihan Guo

**Affiliations:** ^1^ Department of Traditional and Western Medical Hepatology, Third Hospital of Hebei Medical University, Hebei Provincial Key Laboratory of liver fibrosis in chronic liver diseases, Shijiazhuang, China; ^2^ School of Engineering, Penn State Erie, The Behrend College, Erie, PA, United States; ^3^ Shanghai Ashermed Healthcare Co., Ltd., Shanghai, China

**Keywords:** hepatocellular carcinoma, blood biomarkers, machine learning, hepatitis B, cirrhosis, risk model, immunotherapy

## Abstract

Hepatitis B Virus (HBV) infection may lead to various liver diseases such as cirrhosis, end-stage liver complications, and Hepatocellular carcinoma (HCC). Patients with existing cirrhosis or severe fibrosis have an increased chance of developing HCC. Consequently, lifetime observation is currently advised. This study gathered real-world electronic health record (EHR) data from the China Registry of Hepatitis B (CR-HepB) database. A collection of 396 patients with HBV infection at different stages were obtained, including 1) patients with a sustained virological response (SVR), 2) patients with HBV chronic infection and without further development, 3) patients with cirrhosis, and 4) patients with HCC. Each patient has been monitored periodically, yielding multiple visit records, each is described using forty blood biomarkers. These records can be utilized to train predictive models. Specifically, we develop three machine learning (ML)-based models for three learning tasks, including 1) an SVR risk model for HBV patients *via* a survival analysis model, 2) a risk model to encode the progression from HBV, cirrhosis and HCC using dimension reduction and clustering techniques, and 3) a classifier to detect HCC using the visit records with high accuracy (over 95%). Our study shows the potential of offering a comprehensive understanding of HBV progression *via* predictive analysis and identifies the most indicative blood biomarkers, which may serve as biomarkers that can be used for immunotherapy.

## 1 Introduction

Hepatitis B virus (HBV) infection is a worldwide public health crisis. According to the World Health Organization (WHO), 316 million people have chronic HBV infection in 2019, while approximately 555,000 people die from HBV infection globally each year, with hepatocellular carcinoma (HCC) accounting for 45% of deaths (1). Primary liver cancer is a common malignancy worldwide, including HCC, intrahepatic cholangiocarcinoma (ICC), and mixed hepatocellular carcinoma-cholangiocarcinoma (cHCC-CC), of which HCC accounts for 85-90% (2). According to GLOBOCAN 2020 data, liver cancer has the 6th highest annual number of new cases at 905,700, accounting for 8.3% of new cases of all cancers. Due to its poor prognosis, the number of deaths reached 830,000 in 2020, making it the 3rd leading cause of cancer deaths (3). China is one of the regions with high incidence of liver cancer, the annual number of new cases reaches 410,000 and 391,000 deaths, which is 45.3% and 47.1% of the global rate respectively, and is also the 5th most prevalent malignant tumor and the 2nd leading cause of cancer death in China (4).

Most HCC is asymptomatic in its early stages, and most patients are locally advanced or have distant metastases by the time of diagnosis. The main reason for the low long-term survival rate of liver cancer is, first of all, the imperfect risk assessment of early stage of liver cancer, which leads to 70% to 80% of patients being in the middle to late stage at the time of diagnosis (5).

The detection of serum tumor markers can be one of the main methods for early screening and post-treatment efficacy assessment of HCC. The guidelines for the diagnosis and treatment of primary liver cancer (2020) specify AFP as a common and important index for diagnosis of liver cancer and efficacy testing. Liver fibrosis is a tissue repair response to liver damage caused by various pathogenic factors, and the process of abnormal increase or excessive deposition of extracellular matrix during the repair process, as well as the pathological process of developing cirrhosis and liver cancer (6). Studies (7) have shown that liver fibrosis is a reversible process. Therefore, timely and accurate knowledge of fibrosis changes with AFP and levels can help clinicians grasp the trend, regression and prognosis of patients’ disease.

The key to an effective liver cancer surveillance program that provides early diagnosis and improves prognosis is to have simple and accurate tools to identify patients with different liver cancer risks, reduce patient burden, and optimize resource allocation to increase the frequency of surveillance in high-risk groups. Ultimately, individualized patient monitoring of liver cancer risk can be achieved, thereby improving early diagnosis and treatment of liver cancer and ultimately reducing mortality. Retrospective studies also occupy an important position in clinical research and are important for understanding the efficacy of disease therapies and disease regression in the real world.

The immune system plays a crucial role in the development and progression of HCC. Some patients benefit greatly from immunotherapy with checkpoint inhibitors. Adoptive T-cell transfer, vaccination, and virotherapy are other immune strategies being researched, but none of them have shown consistent clinical efficacy as of yet. One of the promising research direction is the identification and validation of predictive biomarkers, which remains a significant challenge in the checkpoint immunotherapy for HCC.

This study gathered real-world electronic health record (EHR) data from the China Registry of Hepatitis B (CR-HepB) database (8). A total of 396 patients with HBV infection at different stages were extracted from CR-HepB, including 1) patients with a sustained virological response (SVR), 2) patients with HBV chronic infection but without further development, 3) patients with cirrhosis, and 4) patients with HCC. Patients in the database have received constant clinical monitoring, yielding multiple visit records that can be utilized to train predictive models. Each of theses records can be represented by a collection of 40 blood biomarkers. Specifically, we develop three machine learning (ML)-based models for three learning tasks, including 1) an SVR risk model for HBV patients *via* a survival analysis model, 2) a risk model to encode the progression from HBV cirrhosis and HCC using dimension reduction and clustering techniques, and 3) a classifier to detect HCC using the visit records with high accuracy (over 95%). Our study shows the potential of offering a comprehensive understanding of HBV progression *via* predictive analysis.

The rest of this paper is organized as follows. Section 3 describes the datasets used in this study and the details of the adopted methods. In Section 4, several experiments are conducted to evaluate our hypothesis. Finally, inSection 5 we discuss the findings, implications, limitations, and future work.

## 2 Material and methods

### 2.1 Dataset

A collection of 396 patients were extracted from the CR-HepB database, including 234 patients with HBV chronic infection and without further development, 90 patients with cirrhosis, and 72 patients with HCC. The inclusion criteria is: 1) age is greater than or equal to eighteen (any gender), 2) with complete hematological results. The exclusion criteria is: 1) patients with biliary obstruction or other factors causing hepatic sludge or hepatic edema, 2) patients who had a liver transplant, 3) patients with drug-induced hepatitis or autoimmune hepatitis. A total of 2400 visit records were gathered from their clinical visit data in between Jan. 2007 and Jan. 2020. Among these records, 1690, 168, and 542 entries were from chronic HBV, cirrhosis, and HCC patients, respectively. **Table 1** shows the stats of the patients divided into the three classes. The average number of visits for HBV, cirrhosis, and HCC patients are 7.22, 1.87, and 7.53, respectively. It is observed that the cirrhosis patients had less visits compared to HBV and HCC patients, which is a factor that may lead to prediction inaccuracy for this class.

**Table 1:** Stats of the dataset.

Class	# patients	# visits
HBV	234	1,690
Cirrhosis	90	168
HCC	72	542
Total	396	2,400

A total of forty blood biomarkers are utilized as features to represent each visit record for a patient. These features include: qualitative HBeAg, quantitative HBeAg, qualitative HBsAG, quantitative HBsAG, qualitative anti-HBc, quantitative anti-HBc, qualitative anti-HBe, quantitative anti-HBe, qualitative anti-HBs, quantitative anti-HBs, Tri-iodothyronine (T3), Tetra-iodothyronine (T4), thyroid-stimulating hormone (TSH), neutrophilic granulocyte (GR), Low-Density Lipoprotein (LDL), prothrombin time (PT), prothrombin activity (PTA), cholesterol (CHOL), total bilirubin (TBI), total protein (TP), drinking alcohol or not, any prior treatment, nucleoside analogues, absolute lymphocyte count (LY), triglycerides (TG), alpha-fetoprotein (AFP), white blood cell count (WBC), albumin (ALB), direct bilirubin (DBI), alkaline phosphatase (ALP), creatinine (Cr), creatine kinase (CK), cholinesterase (CHE), platelet count (PLT), hemoglobin (HGB), time since first visit, Alanine Transaminase (ALT), glutamyl transpeptidase (gamma-GT), Aspartate Aminotransferase (AST), high-density lipoprotein (HDL).

The trial protocol and the implementation of the pilot study were in accordance with the requirements of the Declaration of Helsinki and other regulations. This study is a non-interventional retrospective study. All recipient personally identifiable information were removed or strictly encrypted.

### 2.2 Survival analysis model

The predictive objective in this study is the time to HBV turning negative. Two biomarkers, including HBeAg and HBsAg are utilized for evaluation. We adopt the Random Survival Forest (RSF) model (9) for survival analysis. An RSF adopts ensemble learning to aggregate a collection of decision tree (DT) models that are trained to be de-correlated to increase the model diversity *via* two ways: 1) each tree in the ensemble is trained on a different subset of the original training set, namely, bootstrapping; 2) for each node of a tree, the algorithm only selects a random subset of features and thresholds for split criterion evaluation. Finally, the predicted results of the DTs are aggregated to form the final prediction outcome. The concordance index (C-index) (10) is utilized as the performance metric.

### 2.3 HBV malignant progression analysis

The proposed distance-based method for HBV malignant progression analysis is detailed as follows.

1. Each visit entry of a patient is represented by a vector with forty elements, corresponding to the forty features of the dataset.
2. The geometric centroids for the three classes (i.e., HBV, Cirrhosis, and HCC) are then computed. In a multi-dimensional space, each patient visit is a data point, and patients in the same class tend to be close to each other.
3. Each patient has multiple visit records, yielding multiple data points with temporal characteristic. Thus, by tracing these data points we can find out which direction the patient’s condition heads to. There are two major cases that are of interest:the data points of a patient either head toward or move away from the centroid of HCC. The former case indicates a malignant progression, while the latter may lead to an SVR. The moving distance of the data point series can be quantified and used for risk analysis.
4. As more patients & visits are added to the dataset, the centroids of the three classes can be updated, and the above step can be re-run.

**Figure 1:**
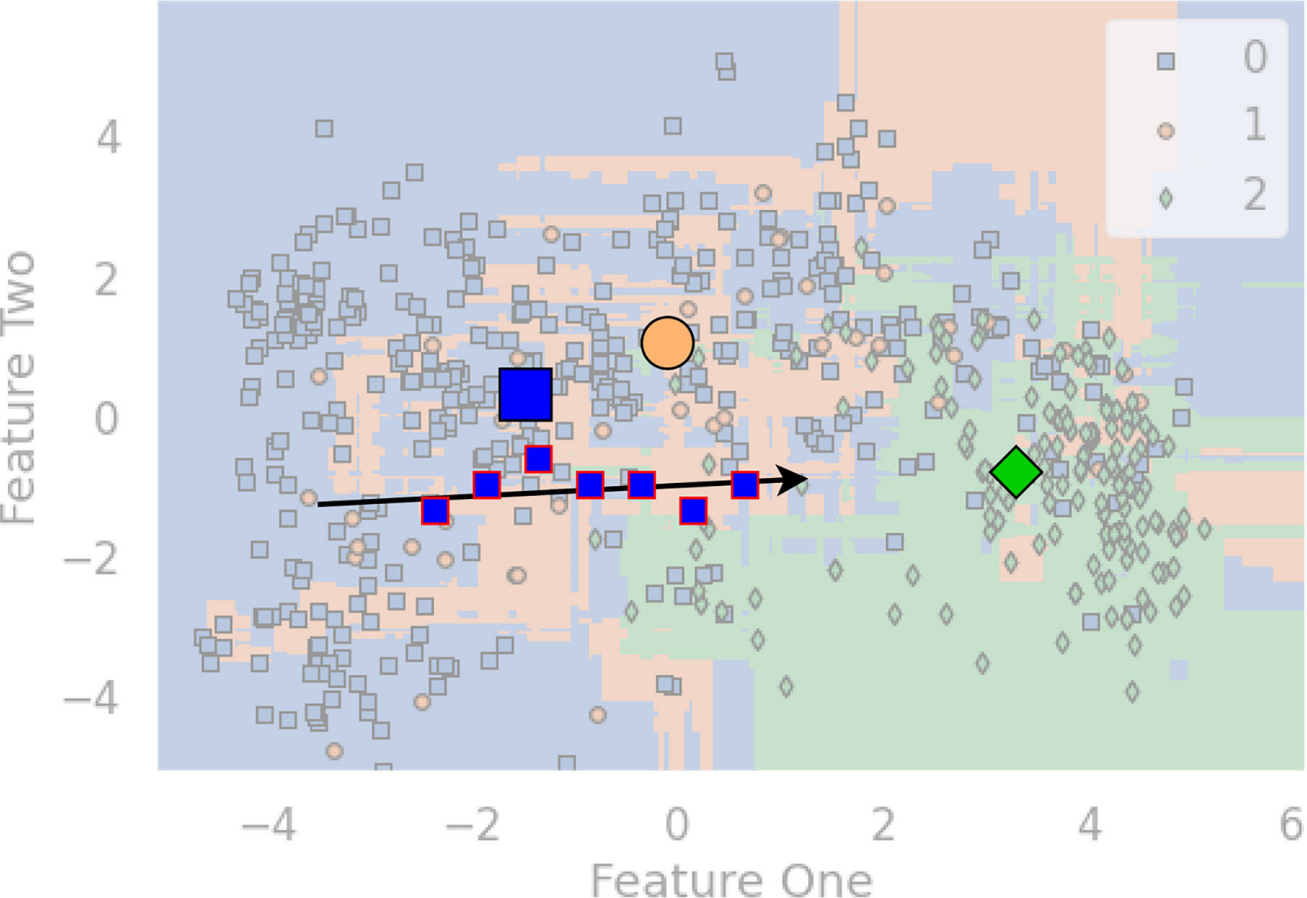
An example diagram that showcases the distance-based method for HBV malignant progression analysis.

**Figure 1** is a diagram that shows an artificial example. To visualize it, we apply principal component analysis (PCA) to project the multi-dimensional features to the 2D space. Patients with HBV, Cirrhosis, and HCC are marked with blue squares, orange circles, and blue diamond shapes. The bigger three shapes represent the three centroids, and a sequence of blue squares with red lines denote the visit records of a patient. Since we want to highlight the example patient, the three centroids, and the arrow, the rest elements of the chart have been moved to the background. The arrow indicates a malignant progression for this patient, since the data points are moving towards the centroid of HCC (ie., the green diamond).

### 2.4 Three-class classification

#### 2.4.1 Predictive models

Fifteen base predictive models were selected to conduct experiments for the classification task. We provide a brief review for these models as follows.

- A Decision Tree (DT) model (11) is trained to be able to construct a tree data structure to make prediction (classification or regression). An internal node of the tree represents a feature, a branch is a learned rule, and a leaf is a predicted outcome. During training, an Attribute Selection Measure (ASM) is utilized to determine which feature is used to split the dataset. The two popular ASMs are information gain (IG) and the Gini index. Taking IG as an example, the training algorithm aims to maximize the overall IG value and takes a greedy method to always select the feature that yields the highest value of IG to build the tree recursively.
- The Random Forest (RF) (12) works by aggregating a collection of DTs, which are a group of weak learners to constitute a more powerful predictive model *via* a voting strategy (13). Since each DT is individually trained, resulting in multiple uncorrelated trees, which helps reduce the variance (14, 15).
- The Adaptive Boosting (ADA) Classifier (16) aggregates a set of weak learners *via* a weighted sum of the individual predicted results. ADA is featured by adjusting the training strategy based on the classification errors, with an aim to fix the misclassified samples and improve the overall predictive performance.
- The Category Boost (CAB) model (17) is also an ensemble model of DTs but focused on gradient boosting. Also, the CAB classifier is featured with a support of categorical features.
- The Extra Trees Classifier (ET) (18) works by fitting a collection of DTs with randomization, namely, the extra trees using a sampling strategy to train these trains with different sampled dataset.
- The Light Gradient Boosting Machine (LGBM) (19) model utilizes a best-first strategy to build a set of DTs, improving the training efficiency compared to the tree-boosting approaches.
- The Gradient Boosting Classifier (GBC) (20) adopts an optimization framework to tackle the boosting procedure by modeling it as a minimization problem on the loss function. Specifically, a set of weak learners, namely, DTs, are used jointly to train the classifier *via* a greedy strategy, i.e., each DT node is split based on the highest score of purity. The overall training algorithm is an additive procedure, involving one DT to be updated in a round, and the rest DTs are unchanged. The goal of each tree addition is to improve the overall predictive accuracy. Once the loss reaches a certain level, the algorithm can be stopped.
- The K Nearest Neighbors (KNN) (21) model adopts a voting strategy from its k-nearest neighbors to determine the prediction outcome of the current data point.
- The Linear Discriminant Analysis (LDA) (22) classifier adopts the Bayes’ rule to derive a a linear boundary by fitting the conditional class densities using the training data. For each class, a Gaussian density is fit so that data points belonging to different classes can be separated.
- The Logistic Regression (LR) (23) model can predict the occurrence probability of an event, using the given datasets with a set of data points in two classes. Although originally designed for binary classification, extension to multi-class classification is straightforward. The model’s outcome is in between 0 and 1. A threshold of 0.5 is usually used to determine positive vs. negative classes when dealing with a classification problem.
- The Naive Bayes (NB) (24) model is based on the Bayes’ theorem. Specifically, a collection of supervised models are trained with an assumption that two features in a feature pair are conditionally independent given the class label.
- The Quadratic Discriminant Analysis (QDA) (22) model is similar to LDA but adopts a quadratic decision boundary to distinguish the data points belonging to two or more classes.
- The Ridge model (25) works by fistly converting the targets from {0, 1} to {-1, 1}, and then treating the task as a regression model to perform a gradient decent optimization.
- The Support vector machine (SVM) (26) with a linear kernel, is a discriminative that is widely used. The model aims to find the optimal hyperplane in a high dimensional space to separate the data points that need to be classified.
- The Extreme Gradient Boosting (XGB) (27) model is an accurate and scalable version of boosting-tree models, with an aim to optimize the computing efficiency and predictive accuracy. XGB also uses regularization to control overfitting (28).

#### 2.4.2 Performance metrics

We adopt the Harrell’s C-index [10] (A.K.A. the concordance index) to evaluate the performance of risk models developed for survival analysis. The intuition is that the risk model will assign a score indicatingthe chance of turning negative for each patient, and the patient with a higher score should have a shorter time-to-turning negative. The computation of C-index involves a pair of patients *i* and *j* (*i* ≠ *j*), with the their scores *s_i_* and *s_j_* and the observed time-to-turning negative *t_i_* and *t_j_*. The patient pair (*i, j*) is said to be a concordant pair if *s_i_* > *s_j_* and *t_i_* < *t_j_*, or it becomes a discordant pair. With this definition, the C-index can be given in Equation 1.


(1) $$\text{C}-\text{index}=\frac{\#\;concordant\;pairs}{\#\;concordant\;pairs+\#\;discordant\;pairs}$$

A value of C-index close to 0.5 means that the predicted score is not better than coin flip to determine which patient’ HBV will turn negative in a shorter period of time. Also, a value close to 1 indicates that the score can well reflect the fact that which patient will be tested negative in HBV first.

For the classification task, we employ five metrics for performance evaluation, including accuracy (Acc), precision (Pre), recall (Rec), F1 score, and Area under the ROC Curve (AUC). Equations 2 - 5 show the definitions of Acc, Pre, Rec, and F1, respectively.


(2) $$Acc=\frac{TP+TN}{TP+TN+FP+FN}$$

(3) $$Pre=\frac{TP}{TP+FP}$$

(4) $$Rec=\frac{TP}{TP+FN}$$

(5) $$F1=2\times \frac{Pre\times Rec}{Pre+Rec}$$

where the terms TP, FP, TN, FN stand for the corresponding numbers of true positives, false positives, true negatives, and false negatives, respectively. The ratio of false alerts is reflected in Pre. The model has less false alerts the higher the pre. Rec displays the number of missed positive samples in the meantime. In other words, the less positive samples that have been overlooked, the higher the Rec. For a classification assignment with an unbalanced dataset, F1 represents the harmonic mean of Pre and Rec and offers a better statistic than Acc. In addition, a ROC curve (receiver operating characteristic curve) is a graph that illustrates the overall performance of a classification model. On a ROC curve, TPR versus FPR are presented for various categorization criteria. When the classification barrier is lowered, more objects are categorized as positive, which increases FP and TP. AUC is the abbreviation for “Area under the ROC Curve.” In other terms, the AUC is the area in two dimensions below the entire ROC curve from (0,0) to (1,1). An overall measure of performance across all potential classification criteria is provided by AUC. AUC is not affected by classification thresholds. Regardless of the categorization threshold that is used, it evaluates the accuracy of the model’s predictions.

## 3 Results

Python 3.7.0 was used to run the experiments for this investigation. PyCaret (29) was adopted to implement the learning methods. Microsoft Office 365 Excel, Matplotlib 3.4.2, and Seaborn 0.11 were used to plot the charts. A self-developed Pythonlibrary named BAIX (https://github.com/aibaix accessed June 9th 2022) was used for data cleaning and exploratory data analysis. For the classification task, the 2,400 samples were divided into a training (1,675) and test (725) set in the ratio of 7:3. A five-fold cross validation (CV) was conducted on the training data.

### 3.1 Results of survival analysis on HBV turning negative

**Figure 2:**
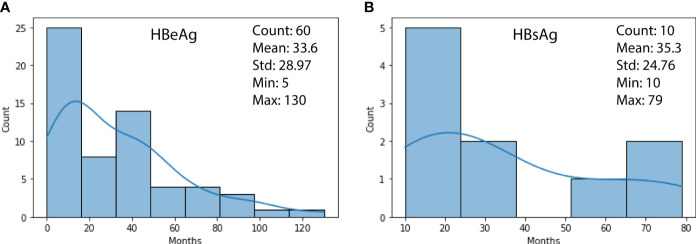
**(A)** Duration distribution of HBeAg turning negative; **(B)** Duration distribution of HBsAg turning negative.

**Figure 2** shows the duration distribution of HBV turning negative for HBeAg (subfigure (a)) and HBsAg (subfigure (b)) biomarkers. It can be observed that 60 patients’ HBeAg have turned negative with a mean of 33.6, a min of 5, and a max of130 months, respectively. For HBsAg, the cases were less (ten patients), with a mean of 35.3, a min of 10, and a max of 79 months, respectively.

**Figure 3:**
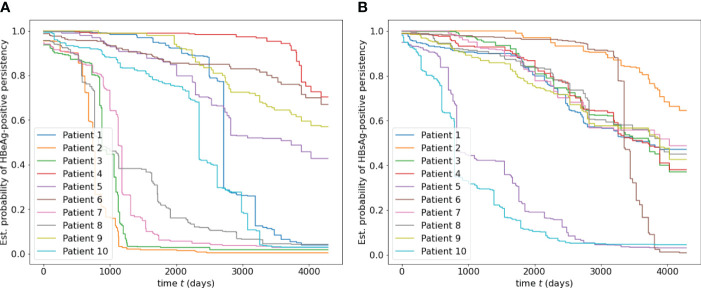
**(A)** Estimated survival functions of HBeAg-positive persistence for ten random patients, with an overall C-index of 0.9104. **(B)** Estimated survival function of HBsAg-positive for ten random patients, with an overall C-index of 0.9075.

**Figure 3** shows two subfigures ((a) and (b)) for the estimated survival functions of HBeAg and HBsAg-positive persistence for ten random patients, with an overall C-index of 0.9104 and 0.9075, respectively. It is observed that different patientspresent different estimated probability of HBV turning negative. In subfigure (a), four patients (2, 3, 7, 8) are more probable to have HBeAg turning negative within 1,000 days, while the rest could take more than 3,000 days. Similar observations can be noted for patients 5 and 10 in subfigure (b). Since the survival function for each individual patient can be plotted and compared with others, a patient and the physician in charge can quickly understand the risk.

### 3.2 Results of malignant progression analysis

**Table 2** displays a three by three matrix that lists the average pair-wise distance between a class of data points to the centroid of the other class. It can be observed that data points in the same class are closer to the centroid of its own class than points from other classes, because the three elements on the diagonal are the smallest vertically and horizontally.

**Table 2:** Distance from to.

From \To	HBV	Cirrhosis	HCC
HBV	1.2517	1.2743	1.5866
Cirrhosis	1.283	1.216	1.4998
HCC	1.3915	1.3329	0.962

**Figure 4:**
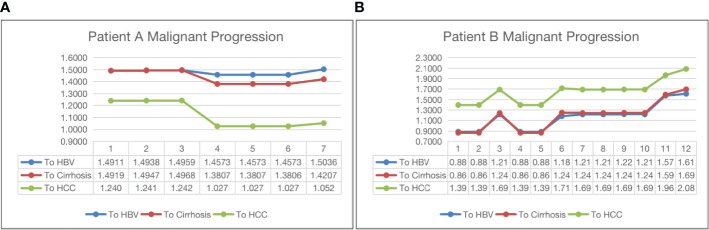
Malignant progression analysis for patient A (subfigure **(A)**) and patient B (subfigure **(B)**).

**Figure 4** shows two opposite examples of HBV progression. Both patients in the two subfigures have been with HBV only. The horizontal axis and the vertical axis represent the visit time and the distance to the centroid of a class, respectively. Subfigure (a) shows that patient A has been moving towards HCC, indicating a malignant progression, because the its distance to the HCC centroid has gradually dropped from 1.24 to 1.052 after seven visits. The distances to HBV and cirrhosis do not change much. On the other hand, in subfigure (b), the patients’ visit records have been moving away from the centroids of all three classes, indicating a potential of SVR.

### 3.3 Results of three-class classification

**Table 3** reports the predictive results of the fifteen models using a five-fold CV. Based on the CV results, we select the best-performing model and evaluate it on the test set. Models in the table are sorted by Acc, and the highest scores foreach metric are marked in bold. We have the following observations.

**Figure 5:**
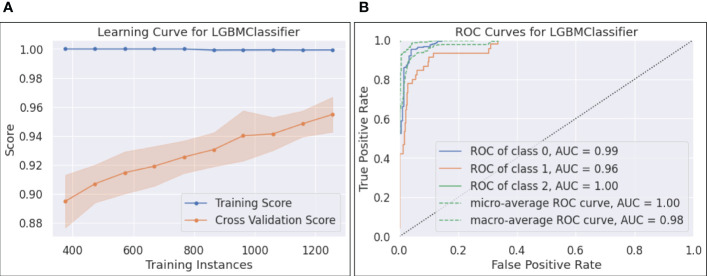
**(A)** Learning curve for LGBM; **(B)** ROC curves for LGBM.

**Figure 6:**
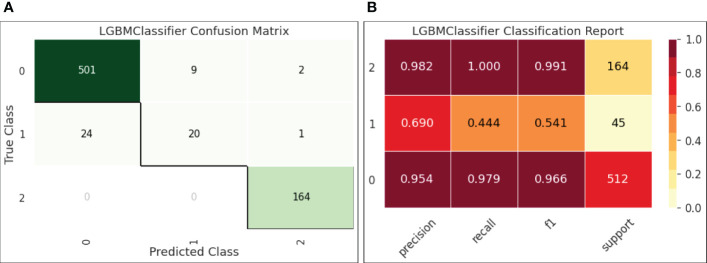
**(A)** Confusion matrix for LGBM; **(B)** Classification report for LGBM.

**Figure 7:**
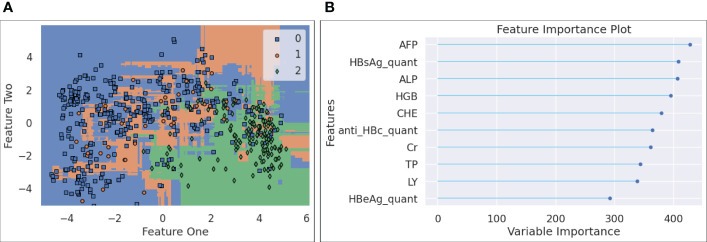
**(A)** Decision boundary chart for LGBM; **(B)** Feature importance chart.

- LGBM presents the highest scores in Acc (0.9548), Pre (0.9511), and F1 (0.9489), the second highest Rec (0.8015), and the third highest AUC (0.9892), making it undoubtedly the best overall model among the fifteen models.
- The other top-five models following LGBM in the table are ET, RF, XGB, and CAB, with an Acc of 0.9516, 0.9503, 0.9497, and 0.9478, respectively. The gaps between LGBM and these models are minor (less than 1%). Another observation is that the top-five models are tree-based, demonstrating the superior modeling ability of tree-based models. Other traditional models, such as SVM, LR, and NB do not fit well on our dataset.
  Based on the results in **Table 3**, the best model, namely, LGBM, is selected for a further evaluation on the test set. Additional results for LGBM are presented in **Figures 5**–**7**. The interpretations of these figures are as follows.
- **Figure 5**-(a) plots the learning curve, which shows the training and CV scores as more instances are utilized in training. It can be seen that the training score reached 1.0 with only 400 training samples, while the CV score was less than 0.9 with the same number of training instances. As more samples participated in training, the training - CV score gap narrowed down, posting a CV score of 0.95, which partially addressed the overfitting issue.
- The ROC curves and the calculated AUCs are shown in **Figure 5**-(b). Five curves, including the ROC curves for each individual class, the micro and macro-average ROC curves, along with the corresponding AUC scores, are reported. Since each ROC curve plots TPR vs. FPR at various classification thresholds, AUC is threshold-invariant. An ideal ROC curve stays to the top left area of the chart, yielding an AUC score close to 1.0. In **Figure 5**-(b), the AUCs for the three classes were 0.99, 0.96, and 1.0, and the micro and macro-average AUCs were 1.0 and 0.98, respectively. The results show that the selected LGBM model was robust and performed well with different thresholds. However, we found that the AUC score of 0.96 for class 1 (i.e., cirrhosis) overstated its performance on this class, reflected by a relatively low F1 for cirrhosis (see 6-(b)).
- **Figure 6**-(a) reports the confusion matrix of LGBM on the test set. The results shown in the matrix are aligned with our observations on the decision boundary chart. Classes 0 (HBV) and 2 (HCC) were well-predicted, while class 1 (cirrhosis) samples were easily classified into class 0, indicating that the current feature setting is effective to separate HBV and HCC but not so effective to distinguish HBV and cirrhosis.
- **Figure 6**-(b) quantifies the results in the confusion matrix with Pre, Rec, and F1 reported for each individual class. It is shown that for class 1 (cirrhosis), both Pre and Rec were low, leading to a low F1 (0.541). Also, the number of samples for cirrhosis was only 45 in the test set. The insufficient samples could be another reason of this under-performance.
- **Figure 7**-(a) plots the decision boundary for the three classes after the forty features were projected to a 2D dimension space. Since LGBM is tree-based, the decision boundary consists of a collection of horizontal and vertical linesegments, attempting to separate the three classes. It is noted that the blue samples (HBV) and green ones (HCC) take different regions within the plotted 2D space, and that the orange ones (cirrhosis) are more difficult to be distinguished, as they are spread across both areas taken by HBV and HCC points. This observation is interesting, since it leads to our hypothesis that if an HBV patient, after multiple visits, finds that a clear visual path can be observed towards the centroid of the HCC area, it may indicate that the risk of HCC has been increasing for this patient.
- The feature importance data are displayed in **Figure 7**-(b), where the top ten most important features are listed and ranked by a variable importance score. The top ten features are AFP, quantitative HBsAg, ALP, HGB, CHE, quantitativeanti HBc, Cr, TP, LY, and quantitative HBeAg.

**Table 3:** Performance comparison.

Model	Acc	AUC	Rec	Pre	F1
LGBM	**0.9548**	0.9892	0.8015	**0.9511**	**0.9489**
ET	0.9516	**0.9913**	0.7954	0.9468	0.9457
RF	0.9503	0.9901	0.7846	0.9474	0.9434
XGB	0.9497	0.9865	0.7961	0.9445	0.944
CAB	0.9478	0.9824	0.7838	0.943	0.9414
GBC	0.942	0.9729	0.7877	0.9387	0.9361
DT	0.9287	0.919	0.7936	0.9259	0.9268
KNN	0.8917	0.9586	**0.8336**	0.9196	0.9013
QDA	0.8783	0.8916	0.6766	0.8869	0.8779
SVM	0.8	0	0.7518	0.8793	0.827
LR	0.7917	0.9296	0.7643	0.8845	0.8228
Ridge	0.7694	0	0.7691	0.8788	0.8011
LDA	0.7592	0.9285	0.7619	0.8727	0.7912
ADA	0.7344	0.8716	0.669	0.852	0.7762
NB	0.7287	0.8962	0.7038	0.8511	0.77

The highest scores for each metric are marked in bold.

## 4 Discussion

Several studies showed the most indicative biomarkers were involved in anti-HBV infection immunotherapy. Secretion of HBsAg and HBeAg promoted macrophage polarization from M1 phenotype towards M2 *via* the SIRT1/Notch1 pathway (30). Inhibitory receptor programmed death receptor 1 (PD-1) which contributes to T cell exhaustion was well-tolerated in chronic hepatitis B virus infection (CHB) HBeAg-negative patients, which caused HBsAg decline in most CHB patients (31). Furthermore, macrophage polarization and T cell exhaustion are both related to tumor immunotherapy, which reminds us biomarkers identified in the study may provide a new potential target for immunotherapy.

This study collected real-world electronic health record (EHR) data from 25 hospitals in China and built a cohort of 480 patients with HBV infection at different stages, including 1) patients with a sustained virological response (SVR), 2) patients with HBV chronic infection and without further development, 3) patients with cirrhosis, and 4) patients with HCC. Each patient has been monitored periodically, yielding multiple visit records that can be utilized to train predictive models. Specifically, we develop three machine learning (ML)-based models for three learning tasks, including 1) an SVR risk model for HBV patients *via* a survival analysis model, 2) a risk model to encode the progression from HBV cirrhosis and HCC using dimension reduction and clustering techniques, and 3) a classifier to detect HCC using the visit records with high accuracy (over 95%). Our study shows the potential of offering a comprehensive understanding of HBV progression *via* a predictive and multi-factor analysis using data with pure blood biomarkers. The most indicative biomarkers identified in the study may serve as biomarkers that can be used for immunotherapy.

The proposed method can be extended in the following directions. First, since the visit records for a patient are sequential, it would be feasible to apply sequential models such as gated recurrent units (GRU), long short-term memory (LSTM), and Bidirectional Encoder Representations from Transformers (BERT) for predictive analysis. Second, this study demonstrate the feasibility of the three predictive tasks applied on a small dataset with only 396 patients. The experiments can be readily extended to a dataset at a large scale.

## Data availability statement

The dataset supporting the conclusions of this article are available upon request. Please contact the corresponding author(s).

## Author contributions

Conceptualization and methodology, YN, SZ, XZ, ZX, and RG; data analysis, software, validation, and original draft preparation, YN, SZ, and XZ; review and editing, and supervision, YN, SZ, XZ, and RG; All authors have read and agreed to the published version of the manuscript.
